# Endohepatology in the Management of Portal Hypertension in Metabolic Dysfunction‐Associated Steatotic Liver Disease

**DOI:** 10.1111/liv.70953

**Published:** 2026-10-26

**Authors:** Michael Praktiknjo, Reiner Wiest, Andres Cardenas, Jonel Trebicka, Wim Laleman

**Affiliations:** ^1^ Department of Medicine B University Hospital Münster Münster Germany; ^2^ Department of Visceral Surgery and Medicine Inselspital, Bern University Hospital, University of Bern Bern Switzerland; ^3^ GI/Liver Unit, Institute of Digestive Diseases & Metabolism Hospital Clínic Barcelona Spain; ^4^ Ciber de Enfermedades Hepáticas y Digestivas (CIBEREHD) Madrid Spain; ^5^ Institut d'Investigacions Biomèdiques August Pi‐Sunyer (IDIBAPS) Barcelona Spain; ^6^ Department of Gastroenterology and Hepatology, Section of Liver and Biliopancreatic Disorders and Liver Transplantation University Hospitals Leuven, KU Leuven Leuven Belgium

**Keywords:** endohepatology, EUS‐PPG, MASH, MASLD, portal hypertension

## Abstract

Portal hypertension is the principal driver of decompensation in advanced chronic liver disease, yet in metabolic dysfunction‐associated steatotic liver disease (MASLD) it deviates from the classical sinusoidal paradigm. Growing evidence points to a mixed sinusoidal–presinusoidal phenotype in which the hepatic venous pressure gradient (HVPG) may underestimate true portal pressure, and in which decompensation and varices can occur at lower HVPG values than in viral cirrhosis. This biology is compounded by a diagnostic gap: non‐invasive tests such as liver stiffness by vibration‐controlled transient elastography, FIB‐4, ANTICIPATE‐NASH, spleen stiffness and the NICER model, are degraded by obesity through technical limitations and altered diagnostic thresholds, even when test discrimination is preserved. Moreover, no non‐invasive rule‐in for clinically significant portal hypertension reaches a 90% positive predictive value in obese MASLD, leaving a clinically meaningful grey zone. Endohepatology addresses these diagnostic limitations by providing complementary hemodynamic, elastographic, and histological information. Within a single sedated session, EUS‐guided shear‐wave elastography and EUS‐guided liver biopsy can adjudicate F2–F3 fibrosis when treatment eligibility is uncertain; EUS‐guided portal pressure gradient measurement can provide a direct gradient when HVPG or non‐invasive tests are discordant with the clinical phenotype; and EUS‐based vascular assessment can characterize collateral and variceal anatomy. The same platform extends to therapy, including EUS‐guided gastric variceal obliteration and pre‐bariatric risk stratification. Current evidence is largely early‐phase and requires prospective validation. Rather than replacing established tools, endohepatology offers an integrated pathway tailored to the diagnostic and therapeutic problems most characteristic of MASLD.

## Lay Summary

- In people with metabolic dysfunction associated steatotic liver disease, obesity and the complex causes of increased portal pressure in the liver can make standard tests less reliable.
- New endoscopic ultrasound‐guided techniques can measure portal pressure gradient, assess liver scarring, obtain tissue samples and evaluate enlarged veins during a single procedure.
- These techniques may be particularly useful when standard tests give uncertain or conflicting results, but further studies are needed to define when they improve patient care.

**Abbreviations**
AUROC: area under the receiver‐operating‐characteristic curve
cACLD: compensated advanced chronic liver disease
CSPH: clinically significant portal hypertension
EASL: European Association for the Study of the Liver
EGD: esophagogastroduodenoscopy
ESG: endoscopic sleeve gastroplasty
EUS: endoscopic ultrasound
EVL: endoscopic variceal ligation
FIB‐4: Fibrosis‐4 Index
GVB: gastric variceal bleeding
HVPG: hepatic venous pressure gradient
LB: liver biopsy
LSM: liver stiffness measurement
MASH: metabolic dysfunction associated steatohepatitis
MASLD: metabolic dysfunction associated steatotic liver disease
MEFIB: MRE + FIB‐4 index
MRE: magnetic resonance elastography
NICER: non‐invasive CSPH estimated risk
NIT: non‐invasive test
NPV: negative predictive value
NSBB: non‐selective beta‐blocker
PPG: portal pressure gradient
PPV: positive predictive value
RCT: randomized controlled trial
SSM: spleen stiffness measurement
SWE: shear wave elastography
TIPS: transjugular intrahepatic portosystemic shunt
VCTE: vibration‐controlled transient elastography

## 1 Introduction

Metabolic dysfunction‐associated steatotic liver disease (MASLD) has become a major cause of advanced chronic liver disease and portal hypertension. Its assessment can be particularly challenging because MASLD itself and associated obesity affect the performance of several non‐invasive tests, while portal hypertension may include both sinusoidal and presinusoidal components. At the same time, advances in therapeutic endoscopy and endoscopic ultrasound (EUS) have expanded the interface between hepatology and interventional endoscopy, giving rise to the emerging field of endohepatology.

This narrative review examines the current and potential role of endohepatology in MASLD, with particular emphasis on clinical scenarios in which conventional diagnostic or therapeutic pathways leave relevant uncertainty. It focuses on EUS‐guided portal pressure gradient measurement (EUS‐PPG), EUS‐based elastography, EUS‐guided liver biopsy, endoscopic and EUS‐based assessment of portal hypertensive manifestations and therapeutic endoscopy for complications of portal hypertension. Their potential integration into a single‐session endohepatology approach and their role in pre‐bariatric assessment are also discussed. Established non‐invasive tests, cross‐sectional imaging, hepatic venous pressure gradient (HVPG) and interventional radiological approaches are considered where relevant as comparators or complementary modalities rather than reviewed comprehensively.

This review focuses specifically on the clinical challenges posed by MASLD. These include the potential contribution of presinusoidal portal hypertension, reduced reliability of several non‐invasive tests in obesity and the increasing need for accurate F2/F3 fibrosis adjudication to guide disease‐specific therapy. Accordingly, we focus on where endohepatology may add clinically relevant information or therapeutic options specifically within the management pathway of patients with MASLD.

For this narrative review, we searched PubMed/MEDLINE for modern era English‐language publications from 2012 to September 2026 addressing MASLD, portal hypertension, endohepatology, EUS‐PPG, EUS‐guided liver biopsy, EUS‐based elastography and endoscopic management of portal hypertension. Relevant original studies, systematic reviews, consensus statements and international guidelines were selected based on their relevance to the clinical questions addressed, supplemented by pertinent references identified from the bibliographies of retrieved articles.

## 2 Pathophysiology of Portal Hypertension in MASLD and the Resulting Diagnostic Gaps

Portal hypertension is the key hemodynamic driver of decompensation in advanced chronic liver disease, but in MASLD its biology appears less straightforward than in viral‐ or alcohol‐related cirrhosis. In the classical model, portal hypertension is predominantly sinusoidal: fibrosis, remodelling with nodule formation, endothelial dysfunction and dynamic intrahepatic vasoconstriction progressively increase intrahepatic resistance while splanchnic vasodilation and hyperdynamic circulation further augment portal inflow. This framework underpins the use of the hepatic venous pressure gradient (HVPG) as the clinical reference standard because wedged hepatic venous pressure usually reflects portal pressure well in predominantly sinusoidal portal hypertension [1].

MASLD, however, seems to deviate from this canonical paradigm. Increasing evidence suggests that portal hypertension in MASLD may arise earlier than expected from fibrosis stage alone and may include a relevant presinusoidal component. Rather than representing only a late consequence of end‐stage architectural distortion, portal hypertension in MASLD may reflect the combined effects of steatosis, hepatocellular ballooning, lobular inflammation, sinusoidal compression, microvascular dysfunction and periportal remodelling. This mixed sinusoidal–presinusoidal phenotype helps explain why some patients develop portal hypertension‐related complications despite apparently ‘sub‐threshold’ HVPG values [2, 3, 4].

The strongest physiologic support for this concept comes from Ferrusquía‐Acosta et al. In patients undergoing TIPS, the agreement between wedged hepatic venous pressure and directly measured portal pressure was excellent in alcohol‐ and HCV‐related cirrhosis, but moderate in MASH cirrhosis. Disagreement occurred in 37.5% of patients with MASH cirrhosis versus 14% of controls and was driven mainly by underestimation of portal pressure. In multivariable analysis, MASH aetiology itself was the only independent factor associated with disagreement, supporting the concept that HVPG may incompletely capture the true hemodynamic burden in MASLD/MASH [2]. Importantly, HVPG retains prognostic value across the steatotic liver disease spectrum: in a recent 696‐patient multicentre cohort, clinically significant portal hypertension (CSPH) independently predicted first decompensation in MASLD, MetALD and alcohol‐related disease alike, with comparable outcomes across subtypes once baseline disease severity was accounted for [5]. This conceptual distinction between indirect sinusoidal pressure assessment by HVPG and direct portosystemic pressure assessment by EUS‐PPG is illustrated in Figure 1.

**FIGURE 1:**
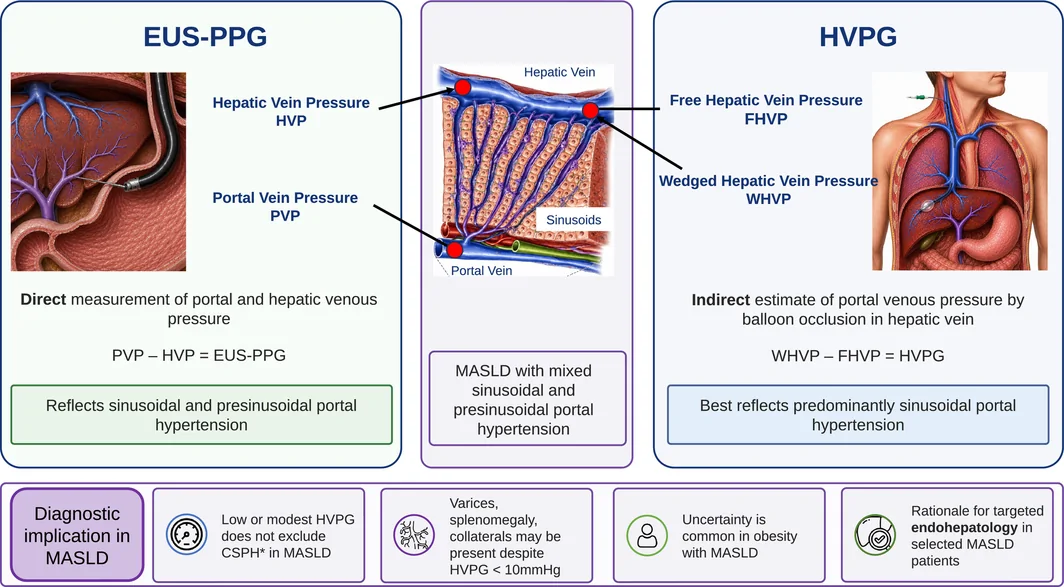
Conceptual comparison of HVPG and EUS‐PPG in MASLD. The figure illustrates the conceptual basis for hemodynamic discordance in MASLD by contrasting hepatic venous pressure gradient (HVPG) with EUS‐guided portal pressure gradient (EUS‐PPG). HVPG is calculated as wedged hepatic venous pressure minus free hepatic venous pressure and therefore provides an indirect estimate of portal pressure that best reflects predominantly sinusoidal portal hypertension. By contrast, EUS‐PPG is calculated as portal vein pressure minus hepatic vein pressure and directly measures the portal pressure gradient. *In MASLD, where portal hypertension may have both sinusoidal and presinusoidal components, low or modest HVPG values do not necessarily exclude clinically relevant portal hypertension, as HVPG‐based criteria for clinically significant portal hypertension (CSPH) may not fully capture presinusoidal portal hypertension. This helps explain why varices, splenomegaly, or collaterals may be present despite HVPG < 10 mmHg, and why targeted endohepatology may be particularly useful in selected MASLD patients.

Clinical outcome data point in the same direction. In the multicentre study by Bassegoda et al. patients with advanced MASLD had lower median HVPG values than patients with advanced HCV‐related disease despite similar liver function, yet showed a higher prevalence of decompensation. For every examined HVPG stratum, decompensation was more frequent in MASLD than in HCV, and some MASLD patients had decompensation and large varices despite HVPG values below 10 mmHg [3]. Differences in comorbidity profiles and definitions of hepatic decompensation across cohorts should also be considered, particularly in MASLD, where cardiovascular and renal comorbidities may complicate attribution of individual clinical events. Thus, HVPG remains prognostic in MASLD, but the same measured value may represent a different hemodynamic reality than in more classically sinusoidal etiologies [1, 2, 3].

This biologic complexity is paralleled by a diagnostic gap in non‐invasive testing. Vibration‐controlled transient elastography (VCTE/FibroScan) is widely used for fibrosis and CSPH risk stratification, but in MASLD, especially with obesity, its performance is less straightforward. Obesity increases technical failure and unreliability largely because of greater skin‐to‐liver capsule distance; the XL probe improves feasibility, but does not eliminate uncertainty, especially in borderline cases [6]. Baveno VII also acknowledged that stiffness thresholds may perform differently across etiologies and body habitus [1]. The ANTICIPATE‐NASH model improved CSPH prediction in compensated MASH cirrhosis by incorporating liver stiffness, platelet count and BMI, but still leaves a clinically meaningful grey zone [7]. This indeterminate group represents the principal potential application of diagnostic endohepatology in MASLD. Endohepatology should not replace validated non‐invasive pathways or HVPG, but may complement them when conventional tests are technically limited, mutually discordant, or inconsistent with the clinical phenotype and when additional information is expected to alter management [2, 3, 8].

In practice, non‐invasive assessment in MASLD is sequential: FIB‐4 is applied first‐line, and patients above the rule‐out threshold proceed to liver stiffness measurement (LSM) by VCTE, interpreted within the Baveno framework of rule‐out, indeterminate and rule‐in zones [1]. The limitations of this pathway are quantitative, not merely conceptual.

For fibrosis staging, VCTE discriminates advanced fibrosis reasonably well (AUROC ~0.77 for ≥ F2 and ~0.80–0.83 for ≥ F3), but its positive predictive value in the treatment‐relevant F2–F3 window is only ~60%–80% and depends strongly on fibrosis prevalence. Importantly, preserved discrimination across BMI categories does not necessarily translate into accurate classification using fixed thresholds. Vali et al. demonstrated comparable AUROCs across BMI subgroups but substantial differences in the liver stiffness thresholds required to achieve predefined diagnostic performance, indicating that clinically relevant misclassification may occur across obesity classes [9, 10, 11]. Additional limitations of non‐invasive diagnostic performance have been demonstrated prospectively. In the LITMUS Imaging Study, no imaging, serum, or composite biomarker significantly exceeded the prespecified minimum acceptable performance criterion for MASH or at‐risk MASH under central histology reading, with NIS2+ the best serum marker at 0.82 [12].

For CSPH, contemporary criteria additionally incorporate spleen stiffness measurement (SSM) and the NICER model (LSM, platelet count, BMI and SSM at 100 Hz), which narrow but do not eliminate the indeterminate zone [13]. Notably, these models discriminate CSPH well across the steatotic liver disease spectrum—ANTICIPATE‐NASH reached an AUROC of 0.83–0.89 across MASLD, MetALD and alcohol‐related disease and outperformed the original ANTICIPATE model [5]—but strong discrimination does not equate to a confident rule‐in.

Crucially, in obese MASLD no non‐invasive rule‐in for CSPH reaches the ≥ 90% positive‐predictive‐value threshold: LSM ≥ 25 kPa is unreliable (~0.67) and even an ANTICIPATE‐NASH probability ≥ 75% reaches only ~0.83 in this population of patients [14]. It is precisely this residual, obesity‐driven grey zone that defines the principal opportunity for diagnostic endohepatology. An integrated non‐invasive pathway for fibrosis and CSPH in MASLD—annotating the diagnostic performance and grey zones of each test and the points at which endohepatology adds value—is proposed in Figure 2, while the subsequent single‐session integration of these endoscopic tools is detailed in Figure 3.

**FIGURE 2:**
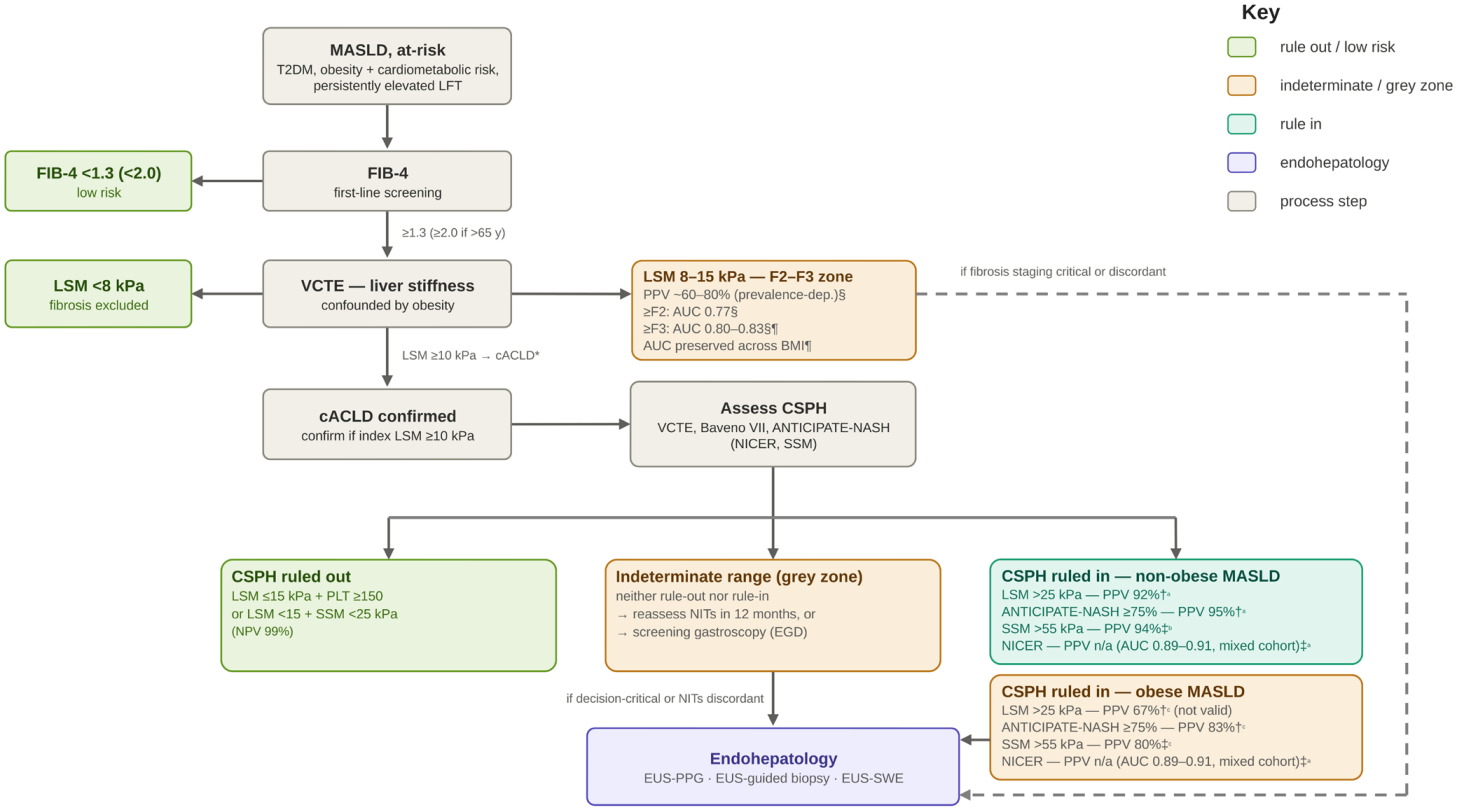
Proposed integrated non‐invasive pathway for fibrosis and clinically significant portal hypertension (CSPH) in MASLD. First‐line FIB‐4 is followed by VCTE‐based liver stiffness measurement (LSM); patients are stratified into rule‐out, indeterminate and rule‐in categories for F2–F3 fibrosis and, once compensated advanced chronic liver disease (cACLD) is confirmed, for CSPH. Positive predictive values (PPV) for CSPH rule‐in are shown separately for non‐obese and obese MASLD because obesity degrades every rule‐in criterion: No non‐invasive rule‐in reaches ≥ 90% PPV in obese MASLD, defining the residual grey zone in which a direct portal‐pressure gradient (EUS‐PPG), EUS‐guided shear‐wave elastography (EUS‐SWE), or EUS‐guided liver biopsy is most likely to change management. *Diagnostic performance*: §, LSM for F2/F3—Eddowes et al. Gastroenterology 2019; ¶, discrimination across BMI and the at‐risk‐MASH ceiling—Vali et al. Lancet Gastroenterol Hepatol 2023 and Liver Int 2025 and Pavlides et al. Nat Med 2026; †, LSM ≥ 25 kPa and ANTICIPATE‐NASH ≥ 75% PPV (overall vs. obese MASLD)—Bañares et al. J Hepatol 2025; ‡, SSM > 55 kPa and the NICER model—Jachs et al. Lancet Gastroenterol Hepatol 2024. *Denominator populations for CSPH rule‐in PPV*: (a) all cACLD (pooled aetiologies); (b) non‐obese cohort (MASLD, viral and MetALD/ALD; *n* = 259); (c) obese MASLD (*n* = 113). Combination non‐invasive tests (Agile 3+; MEFIB) raise rule‐in PPV for significant fibrosis. cACLD and CSPH thresholds follow the Baveno VII framework and its MASLD‐specific refinements. AUROC, area under the receiver‐operating‐characteristic curve; NICER, Non‐Invasive CSPH Estimated Risk; NPV, negative predictive value; NSBB, non‐selective beta‐blocker; SSM, spleen stiffness measurement.

**FIGURE 3:**
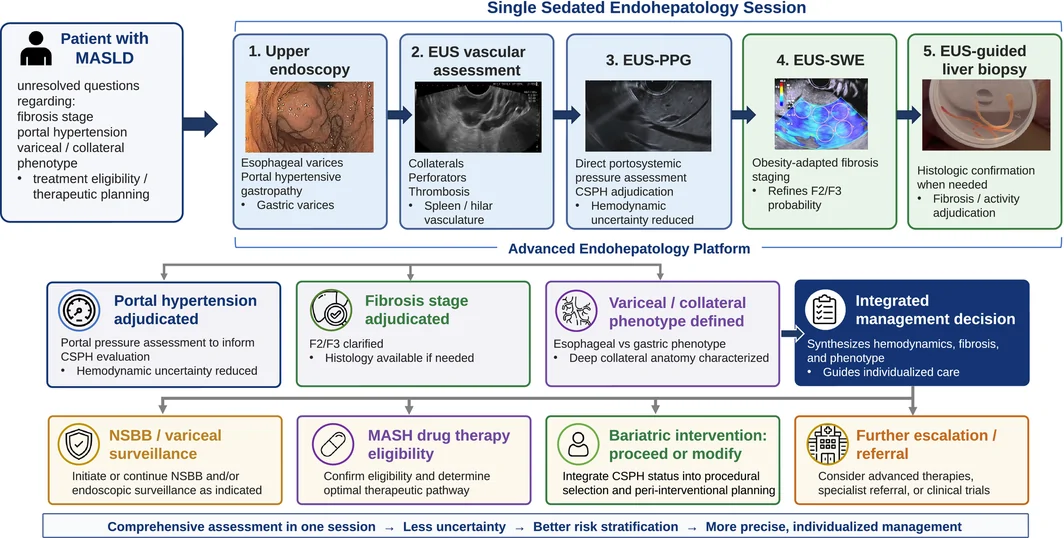
The proposed endohepatology integrated approach in MASLD. In selected patients, a single sedated session can integrate standard upper endoscopy, EUS‐based vascular and collateral assessment, EUS‐guided portal pressure gradient measurement, EUS‐shear wave elastography and EUS‐guided liver biopsy. This modular approach allows simultaneous adjudication of portal hypertension burden, fibrosis stage and variceal/collateral phenotype, thereby generating an integrated management decision regarding prophylaxis, surveillance, treatment eligibility, bariatric risk stratification, or further escalation. This proposed diagnostic pathway has not yet been prospectively validated. EUS‐PPG thresholds for CSPH classification and treatment decisions remain to be established.

## 3 Diagnostic Assessments via Endohepatology in MASLD

### 3.1 Non‐Invasive Fibrosis Staging: EUS‐Guided Shear‐Wave‐Elastography

Fibrosis staging has become treatment‐defining in MASLD/MASH. The FDA and EMA approval of resmetirom and semaglutide for adults with non‐cirrhotic MASH and moderate‐to‐advanced fibrosis, broadly corresponding to F2–F3 stage fibrosis, means that staging now determines not only prognosis, but also indication for therapy [15, 16]. This shift exposes an important limitation of current non‐invasive tests: serum markers and elastography are useful for triage, but often leave a clinically relevant grey zone, particularly in patients with obesity and MASLD. Although VCTE discrimination may remain largely preserved across BMI categories, diagnostic thresholds and predictive values vary with BMI, potentially resulting in misclassification when fixed cutoffs are applied [11]. This limitation is not restricted to Class 3 obesity, although technical failure and overestimation of advanced fibrosis and cirrhosis become particularly relevant in this subgroup [17]. Combination tools that pair elastography with laboratory markers—such as Agile 3+ or the MEFIB index (magnetic resonance elastography plus FIB‐4)—raise rule‐in positive predictive value and can shrink this grey zone before invasive testing is considered [18, 19]. Prospective LITMUS imaging data support this. For advanced fibrosis, magnetic resonance elastography and Agile 3+ both exceeded the acceptability threshold, with AUROC values of 0.91 and 0.84, while serum markers such as FIB‐4 and ELF reached only around 0.74 [12]. Where available, magnetic resonance elastography is therefore a strong non‐invasive modality for fibrosis staging, although performance is lower when the target is at‐risk MASH (AUC 0.75). The specific niche for EUS‐SWE is the obese patient in whom VCTE is technically unreliable and magnetic resonance elastography is not accessible, and in whom the F2 to F3 distinction determines treatment eligibility.

This is where endohepatology becomes particularly attractive. EUS‐guided shear wave elastography (EUS‐SWE) addresses one of the key weaknesses of transabdominal elastography in obesity by shortening the distance of the ultrasound probe to the liver parenchyma from multiple centimetres to less than a centimetre by measurements through the stomach or duodenum. This allows for an optimal region of interest depth of 2 to 4 cm [20]. For VCTE, the recommended skin‐to‐capsula distance (SCD) is < 25 mm for the standard M‐probe and 25 to 35 mm for the XL‐probe. Above 35 mm, reliability of VCTE even with the XL probe decreases [6, 21].

In a pilot tandem study, Kohli et al. showed that EUS‐SWE correlated well with histology and had diagnostic performance comparable to transcutaneous liver stiffness measurement for advanced fibrosis [22]. More importantly for MASLD, Wang et al. conducted a single‐centre pilot study involving 62 patients with obesity and MASLD and found that EUS‐SWE outperformed both FIB‐4 and VCTE for fibrosis staging in this subgroup [23]. Together, these findings support the use of EUS‐SWE where standard elastography is most vulnerable: in patients with obesity and MASLD in whom treatment decisions hinge on whether fibrosis is truly F2/F3.

EUS‐SWE fits naturally into a stepwise endohepatology model primarily as a problem‐solving tool in obese patients with MASLD rather than a replacement for established non‐invasive pathways. In patients with indeterminate non‐invasive tests, it can strengthen confidence that fibrosis is clinically meaningful. If uncertainty persists, the same session can proceed directly to EUS‐guided tissue acquisition (liver biopsy) to clarify treatment eligibility. The strengths, limitations and ideal use cases of the principal diagnostic tools in MASLD‐related portal hypertension are summarized in Table 1.

**TABLE 1:** Diagnostic tools in MASLD‐related portal hypertension: what each tool measures, where it performs best and where its limitations are most relevant.

Tool	What it measures/key cut‐offs	Strengths	Limitations in MASLD/obesity	Best use case	Main unmet issue
EUS‐PPG	Direct portosystemic pressure gradient (EUS) **CSPH:** ≥ 10 mmHg (provisional, extrapolated)^a^	– Direct gradient—May overcome HVPG underestimation—One session with EUS‐LB/SWE/endoscopy	– Advanced EUS required—Limited standardization^a^—Sedation/protocol variation	Discordant HVPG/phenotype; indeterminate NITs; one‐stop work‐up	Standardization; validated cut‐offs
EUS‐SWE	Liver stiffness (EUS) Cut‐offs not standardized (≈ VCTE; under validation)^b^	– Short ultrasound path—Promising in obesity—Same session as other EUS	– Early evidence; limited availability^b^—Less validated than VCTE	Obese MASLD w/unreliable or indeterminate fibrosis tests	Validation; positioning vs. VCTE/biopsy
EUS‐LB	Histology (fibrosis + activity) Stage F0–F4 (reference; no cut‐off)^c^	– Histologic reference—Bilobar sampling; one session—Patient‐preferred	– Invasive; sedation + expertise^c^—Not needed in all patients	Adjudicate F2/F3 eligibility when NITs discordant	Who truly benefits in treatment era
EUS collateral assessment	Deep collaterals, perforators, fundal varices Qualitative; no numeric cut‐off	– Precise vascular phenotyping—Useful for gastric varices	– Limited validation—Operator‐dependent; advanced centres	Recurrent/gastric varices; discordant low HVPG	How findings should change management
Standard upper endoscopy	Luminal varices/portal hypertensive gastropathy Varices small vs. large; red wale = high‐risk^d^	– Direct visualization—Guides EVL and surveillance	– No pressure/fibrosis quantification—Misses deep collaterals	Variceal screening; therapeutic planning	Integrate with hemodynamic/fibrosis data
HVPG	Indirect (sinusoidal) portal‐pressure gradient **CSPH:** ≥ 10 · **Bleeding:** ≥ 12 · **High‐risk:** ≥ 16–20 mmHg^e^	– Gold standard (sinusoidal)—Strong prognostic evidence	– May underestimate portal pressure in presinusoidal aetiology (MASH)^f,g^—Invasive; limited availability	Suspected sinusoidal PH, when expertise available	Interpret ‘low’ HVPG; MASLD thresholds
FIB‐4	Serum fibrosis‐risk score **Rule‐out:** < 1.3 (< 2.0 if > 65 y)^h^ **Rule‐in:** > 2.67	– Inexpensive, widely available—Simple first‐line triage	– Low specificity mid‐range—Not PH‐specific; large grey zone	First‐line population risk stratification	Discriminate F2/F3 & CSPH in individuals
VCTE/FibroScan (LSM + CAP)	Liver stiffness (fibrosis + CSPH) + CAP (steatosis) **Fibrosis:** < 8 kPa rules out significant fibrosis^i^ **cACLD:** < 10 kPa rules out; 10–15 kPa suggestive; > 15 kPa highly suggestive^e^ **CSPH:** ≤ 15 kPa + PLT ≥ 150 rule‐out; ≥ 25 kPa rule‐in (non‐obese)^e^ **CAP:** Steatosis assessment^i^	– Validated, rapid, point‐of‐care—LSM + CAP in one exam	– F2–F3 PPV ~60%–80%^h^—Obesity: thresholds shift, feasibility falls; overestimates in severe obesity^h,j^—Unreliable if SCD > 35 mm (even XL probe)^k,l^	First‐line staging + CSPH rule‐out/in Combine w/Agile 3+, MEFIB to improve rule‐in	Better obese‐MASLD performance; clearer grey zone
MRE	MR‐based liver stiffness assessment Advanced fibrosis: AUROC 0.91 At‐risk MASH: AUROC 0.75^m^	– High accuracy for advanced fibrosis/cirrhosis – Less affected by obesity	– Cost and limited availability – Lower accuracy for at‐risk MASH—Scanner bore and weight restrictions in severe obesity—Iron overload impairs measurement quality—No tissue acquisition or therapeutic capability^m^	Non‐invasive staging of advanced fibrosis/cirrhosis	Defining treatment‐relevant F2/F3 disease/at‐risk MASH
Agile 3+	Composite score combining VCTE‐LSM, AST, ALT, platelets, age, sex and diabetes Advanced fibrosis: AUROC 0.84^m^	– Improves identification of advanced fibrosis compared with FIB‐4 alone—Uses routinely available clinical data	– Requires valid VCTE measurement—Remains an indirect estimate of fibrosis	Non‐invasive identification of advanced fibrosis	Performance in indeterminate/discordant NITs
MEFIB	MRE + FIB‐4 Rule‐in: MRE ≥ 3.3 kPa + FIB‐4 ≥ 1.6^n^	– High rule‐in specificity—Combines imaging and serum assessment	– Requires MRE—Availability and resource requirements	Non‐invasive identification of treatment‐relevant fibrosis	Role relative to newer treatment‐specific algorithms
ANTICIPATE‐NASH	CSPH probability (LSM, platelets, BMI) **Rule‐in:** probability ≥ 75%^o,p^	– MASLD‐adapted CSPH prediction—Improves on single thresholds	– Probability, not diagnosis—Residual grey zone—Obese‐MASLD PPV ~0.83 (< 90%)^p^	Obese/compensated MASLD, uncertain CSPH after VCTE	Turn probabilities into decisions
SSM (spleen stiffness, VCTE 100 Hz)	Spleen stiffness (portal‐pressure surrogate) **Rule‐in:** > 55 kPa **Rule‐out:** < 25 kPa (+LSM < 15)^q^ **Spare EGD:** < 40 kPa^q^	– Portal‐side surrogate (vs LSM)—Same device as LSM; feeds NICER	– Needs 100 Hz spleen module—Overweight‐related failures—Obese‐MASLD rule‐in PPV ~0.80^q^	Refine CSPH indeterminate zone after LSM + platelets	MASLD‐specific validation; reliability criteria; insufficient accuracy for individualized monitoring of NSBB response
NICER (Non‐Invasive CSPH Estimated Risk)	CSPH probability (LSM, platelets, BMI, SSM) Predicted CSPH probability: ≥ 75%^q^ AUROC 0.89–0.91^q^	– High discrimination for CSPH—Adds SSM; point‐of‐care	– Probability, not threshold PPV—Obese‐MASLD data limited; needs SSM^q^—Early adoption	CSPH probability unresolved by LSM + platelets/ANTICIPATE‐NASH	Prospective MASLD validation; thresholds

*Note:* Cut‐offs and diagnostic‐performance: ^a^Vanderschueren et al. *JHEP Rep* 2025, PMID: 40689144; Wang et al. *Clin Gastroenterol Hepatol* 2025, PMID: 41412540; ^b^Kohli et al. *Gastrointest Endosc* 2023, PMID: 36049537; Wang et al. *Gastrointest Endosc* 2025, PMID: 39481576; ^c^Ali et al. *Endosc Ultrasound* 2023, PMID: 37693114; ^d^Gralnek et al. *Endoscopy* 2022, PMID: 36174643; ^e^de Franchis et al. *J Hepatol* 2022, PMID: 35120736; ^f^Ferrusquía‐Acosta et al. *J Hepatol* 2021, PMID: 33068638; ^g^Bassegoda et al. *Clin Gastroenterol Hepatol* 2022, PMID: 34688952; ^h^Vali et al. *Lancet Gastroenterol Hepatol* 2023 and *Liver Int* 2025;45:e16240; ^i^Eddowes et al. *Gastroenterology* 2019, PMID: 30689971; ^j^Velji‐Ibrahim et al. *J Clin Gastroenterol* 2026, PMID: 39787351; ^k^Myers et al. *Hepatology* 2012, PMID: 21898479; ^l^Berger et al. *Clin Transl Gastroenterol* 2019, PMID: 31009403; ^m^Pavlidis et al. *Nat Med* 2026; ^n^Jung et al. *Gut* 2021, PMID: 33685949; ^o^Pons et al. *Am J Gastroenterol* 2021, PMID: 33982942; ^p^Bañares et al. *J Hepatol* 2025, PMID: 41138818; ^q^Jachs et al. *Lancet Gastroenterol Hepatol* 2024;9:1111–1120.

Abbreviations: ALT, alanine aminotransferase; AST, aspartate aminotransferase; AUROC, area under the receiver‐operating‐characteristic curve; BMI, body‐mass index; CAP, controlled attenuation parameter; cACLD, compensated advanced chronic liver disease; CSPH, clinically significant portal hypertension; EGD, esophagogastroduodenoscopy; EUS‐LB, EUS‐guided liver biopsy; EUS‐PPG, EUS‐guided portal pressure gradient; EUS‐SWE, EUS‐guided shear‐wave elastography; EVL, endoscopic variceal ligation; FIB‐4, Fibrosis‐4 index; HVPG, hepatic venous pressure gradient; LSM, liver stiffness measurement; MASLD, metabolic dysfunction‐associated steatotic liver disease; MRE, magnetic resonance elastography; NIT, non‐invasive test; PH, portal hypertension; PLT, platelet count; PPV, positive predictive value; SCD, skin‐to‐capsule distance; SSM, spleen stiffness measurement; VCTE, vibration‐controlled transient elastography.

### 3.2 Invasive Fibrosis Staging: EUS‐Guided Liver Biopsy

Histology remains the reference standard when staging must be definitive or other etiologies for liver disease are considered. This role should be distinguished from prognostic risk stratification. Although models such as ANTICIPATE‐NASH may predict clinical outcomes better than histological fibrosis stage, this does not address the same clinical question when treatment eligibility depends on establishing the presence of F2/F3 fibrosis. Importantly, histological confirmation is not itself required for prescribing resmetirom or semaglutide according to their approved indications.

This is especially true when a patient lies close to the treatment threshold, when non‐invasive tests are discordant, or when long‐term and potentially costly therapy is being considered. EUS‐guided liver biopsy (EUS‐LB) therefore has a targeted role: not biopsy for everyone, but biopsy for patients in whom non‐invasive testing does not provide enough confidence. EUS‐LB has practical advantages in this setting. Standard sedation is routine, the needle path is shorter (especially in obese patients), both lobes can be sampled if necessary and biopsy can be combined with other endoscopic or EUS‐based assessments in one encounter. In a prospective randomized trial, Ali et al. found that EUS‐LB was safe and effective, better tolerated than percutaneous liver biopsy, and associated with a shorter hospital stay [24]. A meta‐analysis by Chandan et al. suggested that percutaneous biopsy may still have somewhat higher overall diagnostic accuracy across studies, but the most recent meta‐analysis including only randomized data showed no significant difference in the quality of obtained liver cores [25, 26]. Needle choice also continues to improve yield; for example, 19‐gauge FNB needles have shown better core acquisition than smaller needles in comparative studies [27]. Modern 19G EUS‐LB can provide specimens meeting established adequacy criteria for histological grading and staging. In a recent randomized trial comparing 19G EUS‐LB with percutaneous liver biopsy, specimen adequacy, defined as a total specimen length ≥ 15 mm and ≥ 8 complete portal tracts, was achieved in 97.8% versus 64.4% of patients, respectively. Median total specimen length was 69 versus 20 mm and the median number of complete portal tracts was 42 versus 9, although EUS‐LB specimens showed greater fragmentation [28].

As with all liver biopsy approaches, histological assessment is also subject to sampling variability and interobserver variability, particularly when distinguishing intermediate fibrosis stages. A cluster‐randomized trial additionally reported significantly less post‐procedural pain and discomfort after EUS‐LB than after percutaneous liver biopsy, supporting EUS‐LB as a potentially patient‐preferred approach [29].

The practical implication is simple. EUS‐SWE and EUS‐LB should be positioned as selective complementary tools rather than routine alternatives to non‐invasive testing. Their clearest potential application is in patients with obesity and discordant or technically unreliable tests when accurate identification of F2–F3 fibrosis is expected to alter treatment. In this way, diagnostic endoscopy does not compete with non‐invasive testing, it helps clarify uncertainty.

### 3.3 Portal Hypertension Staging: EUS‐Guided Portal Pressure Gradient

In MASLD, portal hypertension may arise from both sinusoidal and presinusoidal mechanisms, sometimes before advanced cirrhosis is established. This can make conventional staging imperfect and supports selective use of EUS‐guided portal pressure gradient (EUS‐PPG) as an adjunct to established tools. HVPG remains the reference standard and the benchmark for detection of sinusoidal portal hypertension. The more persuasive role for EUS‐PPG is as a targeted complementary tool in patients in whom HVPG or non‐invasive tests are discordant with the clinical phenotype, technically or logistically difficult to obtain, or physiologically likely to underestimate true portal pressure. This is especially relevant in MASLD, where portal hypertension may have a mixed sinusoidal–presinusoidal component and HVPG may therefore incompletely reflect hemodynamic burden [1, 2, 3].

Conceptually, EUS‐PPG is attractive because it measures the gradient directly from the hepatic as well as portal venous blood compartment. Rather than inferring portal pressure from wedged hepatic venous pressure, the EUS approach punctures a hepatic vein and an intrahepatic portal vein branch under real‐time sonographic guidance, then subtracts hepatic venous pressure from portal venous pressure. The first clinical report was published by Fujii‐Lau et al. followed by the first human pilot study by Huang et al. which demonstrated feasibility and apparent safety using a 25‐gauge needle and compact manometer [30, 31]. In practice, the procedure is usually performed from the stomach, targeting the left or middle hepatic vein and then an accessible left portal vein branch, with the needle traversing liver parenchyma to create a parenchymal cover during withdrawal [32, 33, 34, 35].

Several technical details matter. Currently, there is only one approved commercially available system using a proprietary manometer, and a 25‐gauge FNA‐type needle. However, this system is not available or reimbursed in all regions of the world. Recently, a self‐made, low‐cost alternative using a 22G FNA‐needle and a standard pressure transducer normally used for invasive blood pressure measurement, was validated against the commercial system [36]. This approach may facilitate implementation in resource‐limited healthcare systems, although standardized training, measurement protocols and quality control remain essential.

Pressures are generally measured in triplicate, and careful zeroing, stable scope position and flushing protocols are important because these are low‐flow venous measurements. Sedation is another important variable. Deep sedation, general anaesthesia and mechanical ventilation may affect venous return and intrathoracic pressure, just as they can influence transjugular measurements; standardization of sedation and airway management will therefore be important if EUS‐PPG is to be comparable across centres [37, 38, 39]. The ENCOUNTER trial showed that pressures differ between awake measurement and general anaesthesia, although the magnitude of this difference was modest, generally around 1–2 mmHg [38]. This is particularly relevant in patients with severe obesity, in whom procedural sedation and airway management may be more challenging. Low‐dose midazolam or fentanyl does not affect pressures significantly [39, 40].

The available data suggest that EUS‐PPG is feasible and generally safe in expert hands. In the initial human study, technical success was high and no major safety signal emerged [31]. Subsequent prospective and retrospective series have reported technical success rates around 90%–100%, with mostly low adverse‐event rates [41, 42, 43, 44, 45]. However, dedicated data on technical success in patients with obesity remain limited, as most EUS‐PPG series have not reported outcomes stratified by BMI or MASLD aetiology. Bleeding and infection remain the main theoretical concerns, but small‐calibre needles, transhepatic needle paths and prophylactic antibiotics in many protocols appear to mitigate risk. Available safety data are reassuring. A systematic review and meta‐analysis reported an overall adverse‐event rate of 10.9%, of which 93.7% were mild, corresponding to approximately 0.7% non‐mild adverse events. Abdominal pain was the most frequently reported event and bleeding occurred in 3.6%, while no perforations or procedure‐related deaths were reported [45]. However, these findings are derived predominantly from experienced centres, and their generalizability to routine clinical practice remains uncertain. Patient selection nevertheless remains important. Severe thrombocytopenia or clinically relevant coagulopathy require careful assessment and correction where appropriate, while antithrombotic therapy should be managed according to established recommendations for endoscopic procedures. Portal vein thrombosis is not an absolute contraindication but may affect technical feasibility depending on its extent and location and the availability of a suitable portal venous target. Similarly, altered anatomy requires individualized assessment but does not preclude EUS‐guided PPG or liver biopsy, which have also been reported after liver transplantation [36, 46]. Two prospective studies demonstrated good agreement between EUS‐PPG and HVPG under standardized conditions [37, 38]. However, these studies predominantly included etiologies characterized by sinusoidal portal hypertension. More recently, the prospective EVADIPP study evaluated paired EUS‐PPG and HVPG measurements in 90 patients enriched for diseases with a presinusoidal component, including PSVD (35.6%) and MASLD (37.8%). Overall agreement was poor (ICC 0.08), with EUS‐PPG values systematically higher than HVPG. Agreement was only slight in MASLD (ICC 0.23), whereas it was substantial in alcohol‐ and viral‐related liver disease (ICC 0.72). During a median follow‐up of 394.5 days (IQR 231.3–705.0), 28 patients (31%) developed hepatic decompensation. EUS‐PPG demonstrated superior prognostic discrimination compared with HVPG (C‐index 0.78 vs. 0.56) and remained independently associated with decompensation after adjustment for albumin and platelet count (HR 1.19, 95% CI 1.10–1.30). In the MASLD subgroup (*n* = 34, 13 events), this association persisted after adjustment for platelet count (HR 1.19, 95% CI 1.04–1.35). Physiologically, direct measurement of portal venous pressure incorporates resistance upstream of the sinusoidal compartment that may not be fully reflected by HVPG. These preliminary findings suggest that EUS‐PPG may provide additional prognostic information in MASLD [47]. Observational studies have also reported strong associations between EUS‐PPG and clinical manifestations of portal hypertension, including gastroesophageal varices [44, 48]. The available evidence therefore supports EUS‐PPG as a feasible adjunct in expert centres, while thresholds for defining CSPH, guiding treatment and predicting clinical outcomes require further prospective validation.

In MASLD, EUS‐PPG is particularly compelling when clinical signs of portal hypertension seem disproportionate to HVPG, when non‐invasive tests are indeterminate, or when an endoscopic procedure is being performed anyway and direct hemodynamic information is expected to alter management. In that setting, EUS‐PPG fits naturally into a broader diagnostic pathway [2, 3]. The Baveno VIII consensus recognizes EUS‐guided direct portal pressure measurement as an emerging technique and includes its further evaluation within the research agenda for portal hypertension [49]. The key issue is no longer whether EUS‐PPG is technically feasible, but how it can be integrated reproducibly into routine advanced endoscopy practice. In many centres, advanced EUS expertise is likely more accessible than dedicated HVPG expertise, which strengthens the practical appeal of this approach. Institutional protocols should specify venous targets, pressure‐transducer calibration, repeated measurements, sedation strategy, antibiotic prophylaxis and post‐procedural monitoring and should remain aligned with emerging international consensus recommendations [38, 39, 50].

### 3.4 EUS Assessment of Variceal Anatomy, Collaterals and Perforators

Beyond simply identifying varices, endoscopy refines risk stratification by defining variceal size, red wale markings, portal hypertensive gastropathy and the possible presence of coexisting gastric varices. This remains clinically relevant even in the era of non‐invasive CSPH assessment, because many MASLD patients fall into indeterminate zones in which management still depends on direct visualization. In addition, endoscopic findings may help contextualize discordant hemodynamic data; for example, the presence of large varices or marked portal hypertensive gastropathy despite apparently modest HVPG values should raise suspicion that portal hypertensive burden is being underestimated and should trigger further workup.

EUS adds a diagnostic layer that neither HVPG nor standard luminal endoscopy can provide: more precise vascular phenotyping of portal hypertension. It can detect paraesophageal veins, periesophageal collateral veins, perforating veins and deep fundal collaterals that may not be fully appreciated on forward‐viewing endoscopy. These findings are clinically relevant because large perforating veins and severe para−/periesophageal collaterals have been associated with recurrence after endoscopic variceal ligation and may identify patients at higher risk of future bleeding or early variceal return [51, 52]. A meta‐analysis likewise found that perforating veins and periesophageal collateral veins were associated with a higher risk of oesophageal variceal recurrence [53, 54]. However, identification of those features via EUS requires specialty training, and standardization and quantification of those veins is still in development.

This assessment may be particularly informative when the clinical portal‐hypertension phenotype appears disproportionate to the measured pressure gradient. Extensive collateralization despite a modest HVPG may help explain the apparent hemodynamic mismatch and prompt closer surveillance or multidisciplinary reassessment. The most established application of EUS vascular assessment is the characterization of fundal varices, feeder vessels and deeper collateral networks that may not be fully defined by forward‐viewing endoscopy [55, 56, 57]. By contrast, the use of EUS‐detected oesophageal collaterals alone to determine surveillance intervals or initiate treatment remains insufficiently validated.

Contrast‐enhanced CT and MRI provide complementary non‐invasive mapping of the portal and splanchnic vascular anatomy, including portosystemic shunts and collaterals, whereas EUS enables high‐resolution assessment of target vessels with the possibility of therapeutic intervention during the same procedure.

### 3.5 Endohepatology Synergy: Integration of Fibrosis, Pressure and Variceal Assessment

The central clinical proposition of endohepatology in MASLD is that several unresolved questions can be addressed within a single coordinated session: Does the patient truly have CSPH? Is fibrosis severe enough to justify therapy? What is the actual variceal and collateral phenotype? The value of endohepatology lies not in any single test, but in the integration of upper endoscopy, EUS‐PPG, EUS‐SWE, EUS‐guided liver biopsy and targeted spleen or collateral assessment within one sedated session [33, 39, 42, 58]. This integrated concept is summarized in Figure 3.

This integrated approach is attractive because MASLD often presents with diagnostic fragmentation: VCTE may be unreliable or indeterminate in patients with severe obesity [17], HVPG may underestimate portal pressure in selected patients [2] and treatment decisions increasingly depend on accurate characterization both fibrosis stage and portal hypertension. Current Baveno VIII recommendations support predominantly non‐invasive risk stratification in compensated advanced chronic liver disease (cACLD), while ANTICIPATE‐NASH refines CSPH risk estimation and may also stratify the risk of decompensation more accurately than histology in patients with advanced fibrosis [8]. Nevertheless, probability estimates do not always resolve individual clinical decisions. Standard upper endoscopy therefore remains important when variceal status will change management, and EUS can extend the assessment by characterizing paraesophageal and paragastric collaterals, perforating vessels and fundal variceal anatomy [30, 59, 60].

Within the same session, EUS‐PPG provides direct hemodynamic information in selected patients, helping inform decision making on whether CSPH is present and whether primary prophylaxis with NSBBs should be considered [47, 61], while EUS‐SWE and EUS‐guided biopsy address fibrosis staging. In practical terms, the session can move stepwise from screening to confirmation: if EUS‐SWE strengthens suspicion of actionable F2/F3 disease, biopsy can follow immediately; if not, the evaluation can remain less invasive. Spleen and collateral assessment add complementary phenotyping, especially when gastric varices are present or suspected [57].

Not every patient needs the full spectrum of assessments. It is most useful when several decisions are simultaneously pending and when standard pathways leave uncertainty. In that context, one sedated session may reduce the need for multiple visits and diagnostic delays while generating a coherent hepatologic and endoscopic phenotype from a single encounter. However, these potential benefits in clinical efficiency have not yet been demonstrated in comparative studies. In patients with Class 3 obesity, anesthesiologist‐supported sedation or general anaesthesia may be required, potentially increasing procedural complexity and resource utilization. Importantly, simultaneous EUS‐PPG and EUS‐LB has also been evaluated prospectively, supporting the feasibility and safety of combining these procedures within a single endohepatology session [42].

The clinical scenarios in which these diagnostic modalities may resolve uncertainty after conventional assessment and influence subsequent management are summarized in Table 2 and integrated into the proposed pathways in Figures 2 and 3.

**TABLE 2:** Proposed clinical scenarios in which endohepatology may add value in MASLD.

Clinical scenario	Why standard pathway is insufficient	Endohepatology component	Management decision informed by result	Conventional alternative	Evidence status
Obese MASLD + indeterminate NITs	FIB‐4 and VCTE often leave a grey zone; obesity reduces elastography feasibility and specificity; ANTICIPATE‐NASH and the SSM‐based NICER model refine CSPH prediction, but no non‐invasive rule‐in reaches ≥ 90% PPV in obese MASLD, so the individual case may remain unresolved	Upper endoscopy ± EUS‐SWE, EUS‐PPG and in selected cases EUS‐LB	Clarifies whether the patient can be managed non‐invasively, needs portal hypertension prophylaxis, or requires fibrosis adjudication for treatment planning	Repeat/combined NITs; MRE; HVPG where portal pressure adjudication is required	Emerging; MASLD‐specific data available for EUS‐SWE and EUS‐PPG, but integrated pathways remain unvalidated^a^
Uncertain F2/F3 drug eligibility	Borderline or discordant serum markers/elastography may not confidently distinguish non‐actionable fibrosis from treatment‐relevant F2/F3 disease	EUS‐SWE as an obesity‐adapted staging step; EUS‐LB for definitive histologic adjudication when needed	Supports or excludes eligibility for MASH‐directed pharmacotherapy and avoids undertreatment or overtreatment	Repeat/combined NITs; MRE; percutaneous liver biopsy	EUS‐LB established for tissue acquisition; MASLD‐specific EUS‐SWE evidence remains limited^b^
Discordant HVPG and clinical phenotype	HVPG may underestimate true portal pressure in MASLD because of a mixed sinusoidal–presinusoidal pattern; phenotype may suggest greater portal hypertensive burden than the measured gradient	EUS‐PPG ± EUS collateral assessment	May inform whether CSPH is truly present and helps position the patient for NSBBs, endoscopic surveillance/intervention, or escalation strategies	Repeat HVPG; cross‐sectional imaging; clinical/endoscopic reassessment	Prospective MASLD‐specific evidence supports discordance and prognostic value; management thresholds remain to be validated^c^
Varices or splenomegaly with low HVPG	HVPG < 10 mmHg may be falsely reassuring in MASLD when collaterals, thrombocytopenia, splenomegaly, or varices suggest clinically significant portal hypertension	Upper endoscopy, EUS‐PPG, EUS assessment of collaterals/perforators	Reconciles the mismatch and may support initiation of prophylaxis, closer surveillance, or referral for decompressive therapy	Cross‐sectional imaging; repeat HVPG; conventional upper endoscopy	Physiologically plausible with emerging direct PPG evidence; prospective management validation lacking^c^
Pre‐bariatric assessment in compensated MASLD cirrhosis	The safety and choice of bariatric intervention change substantially when CSPH is present. Obesity may limit non‐invasive test performance, and HVPG may underestimate portal hypertensive burden in selected patients with MASLD	Upper endoscopy ± EUS collateral assessment; EUS‐PPG in selected patients with indeterminate or discordant findings; EUS‐SWE/EUS‐LB	Supports proceeding with, modifying, or deferring bariatric intervention and identifies patients requiring multidisciplinary management at an expert centre	NITs/MRE; CT/MRI; HVPG; conventional upper endoscopy	Proposed integrated application; individual components supported, but the combined pathway has not been prospectively validated^d^
Suspected gastric varices/complex collateral anatomy	Standard luminal endoscopy may underestimate deep fundal anatomy, feeder vessels, or collateral complexity	EUS collateral and variceal assessment ± therapeutic planning during the same session	Refines risk stratification and helps select EUS‐guided therapy, conventional endoscopic therapy, or multidisciplinary radiologic escalation	Contrast‐enhanced CT/MR venography; conventional upper endoscopy; interventional radiology assessment	Established EUS imaging/therapeutic application; MASLD‐specific comparative data limited^e^

*Note:* Evidence citations: ^a^Wang et al. *Gastrointest Endosc* 2025; Santopaolo et al. *Endoscopy* 2026. ^b^Wang et al. *Gastrointest Endosc* 2025; Ali et al. *Endosc Ultrasound* 2023. ^c^Santopaolo et al. *Endoscopy* 2026. ^d^European Association for the Study of the Liver. *J Hepatol* 2025; Abad et al. *Clin Gastroenterol Hepatol* 2025. ^e^Mohammadpour et al. *J Gastroenterol Hepatol* 2026; Florencio de Mesquita et al. *Gastrointest Endosc* 2025.

Abbreviations: CSPH, clinically significant portal hypertension; EUS‐LB, EUS‐guided liver biopsy; EUS‐PPG, EUS‐guided portal pressure gradient; EUS‐SWE, EUS‐guided shear‐wave elastography; FIB‐4, Fibrosis‐4 index; HVPG, hepatic venous pressure gradient; MASH, metabolic dysfunction‐associated steatohepatitis; MASLD, metabolic dysfunction‐associated steatotic liver disease; MRE, magnetic resonance elastography; NICER, Non‐Invasive CSPH Estimated Risk; NIT, non‐invasive test; NSBB, non‐selective beta‐blocker; PPV, positive predictive value; SSM, spleen stiffness measurement; VCTE, vibration‐controlled transient elastography.

## 4 Therapeutic Endohepatology in MASLD

### 4.1 Endoscopic Variceal Band Ligation

The management of oesophageal varices in MASLD follows the same general principles as in other etiologies of cirrhosis. Standard upper endoscopy remains the therapeutic gateway when endoscopic assessment is indicated, allowing identification of high‐risk varices and immediate endoscopic variceal ligation (EVL). According to current Baveno VIII recommendations, EVL is particularly relevant for primary prophylaxis when NSBBs are contraindicated or not tolerated, while secondary prophylaxis generally combines NSBBs and EVL [49].

In MASLD, EVL may have particular practical importance because these patients often have metabolic and cardiovascular comorbidities that complicate NSBB use. Hypotension, chronotropic intolerance, renal vulnerability and frailty may all narrow the therapeutic window for NSBBs in individual patients. In such cases, EVL is not merely an alternative technical option, but a clinically important component of individualized primary prophylaxis. At the same time, the endoscopic phenotype should not be interpreted in isolation; patients with recurrent varices, rapid recurrence after ligation, or signs of a high collateral burden may warrant closer follow‐up and earlier consideration of escalation strategies.

### 4.2 EUS‐Guided Gastric Variceal Therapy

Where endohepatology adds the most distinctive therapeutic value is in gastric varices. These bleed less often than oesophageal varices, but when they do, bleeding is often more severe and technically more difficult to manage. Current guidelines recognize cyanoacrylate‐based endoscopic obliteration as a key option and emphasize multidisciplinary decision‐making with radiology and hepatology, especially for GOV2, IGV1 and complex collateral anatomy [49, 62].

EUS has been particularly transformative here. By directly visualizing the varix and its feeder vessels, EUS‐guided therapy allows targeted deployment of coils, glue, or both. Combination therapy is especially effective because the coils provide a scaffold that reduces the amount of glue needed for complete obliteration and therefore may reduce embolization risk. The most recent meta‐analyses found that EUS‐guided coil plus glue was associated with lower reintervention, lower rebleeding, fewer overall adverse events and higher variceal obliteration than other endoscopic modalities, although the certainty of evidence ranged from very low to moderate [59, 60]. However, the general concept of EUS‐guided therapy improving anatomic precision, permitting feeder‐vessel targeting, may be particularly useful in fundal varices, recurrent varices, or cases of incomplete prior glue injection [57, 63, 64].

Therapeutic endoscopy for gastric varices should not be framed as competing with NSBBs or TIPS. NSBBs remain foundational for primary prophylaxis. Therapeutic endoscopy should rather be embedded in a definitive management strategy, not viewed as the endpoint of care. The therapeutic endohepatology pathway for acute and definitive management of variceal bleeding is outlined in Figure S1.

In the setting of acute gastric variceal bleeding, conventional endoscopic glue therapy is advised if EUS guided therapy is not available. Because the risk of rebleeding after initial haemostasis is high, patients with gastric variceal haemorrhage should undergo definitive secondary therapy once bleeding has been controlled. A randomized trial by Biswas et al. showed that, after initial haemostasis with cyanoacrylate, a definitive radiologic strategy with TIPS or BRTO markedly reduced 1‐year gastric variceal rebleeding compared with repeated endoscopic glue therapy alone, and also reduced gastric variceal rebleeding‐related mortality [65]. More recently, the GAVAPROSEC trial showed that in acute bleeding from fundal varices, pre‐emptive TIPS significantly improved 1‐year freedom from rebleeding or death and should be considered first‐line therapy in appropriate candidates [66]. Accordingly, TIPS should be regarded as the preferred definitive treatment for many patients with bleeding fundal gastric varices, not merely as rescue therapy. When TIPS is not feasible, such as in extensive splanchnic venous thrombosis, or is contraindicated, EUS‐guided coil plus glue represents a reasonable alternative definitive treatment, offering targeted complete obliteration under direct vascular visualization.

In selected patients with favourable anatomy, particularly those with accessible spontaneous shunts, additional transvenous strategies such as BRTO or anterograde embolization via transsplenic or transmesenteric access may provide another definitive option, underscoring why multidisciplinary triage with endoscopy, hepatology and interventional radiology is essential [67, 68, 69, 70]. The principal access routes for definitive gastric variceal therapy are illustrated in Figure S2.

### 4.3 Bariatric Interventions

Obesity treatment is central to MASLD management, but cirrhosis and clinically significant portal hypertension substantially alter the risk–benefit balance of bariatric intervention. Current guidance supports consideration of bariatric surgery only in carefully selected patients with compensated cirrhosis without CSPH and in experienced multidisciplinary centres [71]. Accurate assessment of portal hypertension is therefore an essential component of pre‐bariatric evaluation.

This issue is particularly relevant in MASLD because obesity can reduce the reliability of transabdominal elastography, while HVPG may underestimate portal hypertensive burden in patients with a mixed sinusoidal–presinusoidal phenotype. In patients with indeterminate non‐invasive tests, discordant clinical findings, or suspected portal hypertension despite a low HVPG, targeted endohepatology may help resolve pre‐procedural uncertainty. Upper endoscopy can identify varices and portal hypertensive gastropathy, EUS can characterize collateral anatomy and EUS‐PPG may provide direct hemodynamic assessment when the result is expected to determine whether bariatric intervention can proceed [2, 3, 23, 38].

Endoscopic bariatric therapies may offer an alternative for selected patients who are not suitable candidates for surgery.

Several endoscopic metabolic and bariatric therapies are available or under investigation, including endoscopic sleeve gastroplasty [72, 73, 74, 75], intragastric balloons and small‐bowel interventions [76]. These approaches can achieve clinically relevant weight loss and improvements in metabolic parameters, making them potentially attractive in selected patients with MASLD [77, 78, 79, 80]. Within an endohepatology pathway, their particular relevance lies in the possibility of integrating metabolic intervention with assessment of fibrosis and portal hypertension to inform patient selection and procedural risk. However, direct evidence for bariatric endoscopy in patients with established portal hypertension remains limited, and no endoscopic bariatric intervention has yet been shown to reduce portal pressure or improve portal hypertension‐related clinical outcomes. Prospective studies in this specific population are therefore needed.

## 5 Clinical Algorithms and Future Directions

A practical endohepatology algorithm in MASLD should begin with the recognition that the greatest unmet need lies not in obvious low‐ or high‐risk patients, but in those with diagnostic discordance. In patients with obese MASLD and indeterminate non‐invasive tests, the first step remains structured triage with FIB‐4, platelets and liver stiffness, interpreted within Baveno VIII and MASLD‐specific refinements such as ANTICIPATE‐NASH (Figure 2) [8, 49]. However, obesity reduces the reliability and specificity of elastography‐based pathways, and although MASLD‐adapted models—ANTICIPATE‐NASH and the SSM‐based NICER model—refine CSPH prediction, no non‐invasive rule‐in reaches ≥ 90% positive predictive value in obese MASLD, so a clinically meaningful grey zone persists [14]. In this group, endoscopy is often needed anyway; a rational next step is combined diagnostic endohepatology with upper endoscopy and, when the result would alter management, EUS‐SWE, EUS‐PPG and/or EUS‐LB [33, 34, 58]. Proposed clinical scenarios in which endohepatology may add value in MASLD are summarized in Table 2.

A second pathway concerns exclusion of CSPH before bariatric intervention. Because the safety and selection of bariatric procedures change substantially once CSPH is present, patients with compensated MASLD cirrhosis and uncertain pre‐procedural risk represent a focused use case for diagnostic endohepatology. When non‐invasive tests are indeterminate or discordant with the clinical phenotype, upper endoscopy and, in selected patients, EUS‐PPG may help determine whether bariatric intervention can proceed, should be modified, or should be deferred [2, 3, 71].

A third pathway involves uncertainty about F2/F3 drug eligibility. With resmetirom and semaglutide now approved for MASH with moderate‐to‐advanced fibrosis, EUS‐SWE and, when needed, EUS‐guided (potentially bilobar) liver biopsy provide a way to adjudicate fibrosis stage when non‐invasive tests are borderline or discordant [15, 16, 23, 24].

For example, a patient with obesity and MASLD may have an indeterminate FIB‐4 score and discordant VCTE results, leaving uncertainty about eligibility for MASH‐directed therapy. EUS‐SWE may provide additional information, while EUS‐guided liver biopsy can establish the fibrosis stage when uncertainty persists. If biopsy confirms F2–F3 fibrosis and the other treatment criteria are met, MASH‐directed pharmacotherapy may be considered. Conversely, the absence of significant fibrosis could prevent unnecessary treatment.

A fourth pathway concerns discordant HVPG and clinical signs, or varices and splenomegaly with low HVPG. In MASLD, low or ‘non‐CSPH’ HVPG should not automatically overrule a phenotype that includes collaterals, thrombocytopenia, splenomegaly, varices, or decompensating events. In such patients, EUS‐PPG and EUS assessment of collateral anatomy may help reconcile the mismatch and better position the patient for NSBB therapy, endoscopic surveillance or intervention, or therapeutic referral.

Looking ahead, the priority is no longer to establish that endohepatology is feasible, but to define how it can be used most effectively in MASLD or otherwise said endohepatology adds another arrow to the hepatologist's bow; the art lies in knowing when to use it.

Given that only around half of patients achieve an adequate hemodynamic response to NSBB therapy, serial EUS‐PPG before and after NSBB initiation represents an attractive potential application of direct portal pressure measurement. Such a pressure guided approach using HVPG has shown favourable outcomes in secondary prophylaxis [81] and may spare unnecessary NSBB‐related adverse events and trigger therapy escalation in non‐responders. However, this approach has not yet been prospectively validated. Future work should standardize EUS‐PPG methodology and clinically relevant thresholds, clarify where one‐stop endohepatology offers the greatest advantage over sequential conventional testing, and translate these tools into practical algorithms for fibrosis adjudication, portal hypertension assessment and therapeutic planning. Specifically, studies should establish test–retest reproducibility under standardized measurement and sedation conditions, define the magnitude of change that exceeds procedural and physiological variability. Prospective studies in MASLD should compare paired EUS‐PPG and HVPG measurements against independently measured direct portal pressure, obtained during TIPS placement or through transhepatic access, and assess their respective associations with subsequent hepatic decompensation. Moreover, Comparative studies should also assess resource utilization and cost‐effectiveness of integrated endohepatology pathways versus sequential conventional testing, recognizing that these parameters are likely to vary substantially between healthcare systems.

In a disease marked by obesity, diagnostic grey zones and hemodynamic discordance, endohepatology is particularly well suited to move from proof of concept toward precision clinical application.

## 6 Conclusion

MASLD highlights both the value and the limitations of current portal hypertension assessment. HVPG and non‐invasive tests remain foundational, but their performance is less straightforward in a disease characterized by obesity, diagnostic grey zones and a likely mixed sinusoidal–presinusoidal pattern of portal hypertension [2, 3]. In this setting, endohepatology offers a coherent framework: EUS‐PPG can clarify hemodynamic uncertainty, EUS‐SWE and EUS‐guided liver biopsy can refine fibrosis staging when treatment eligibility depends on identifying F2/F3 fibrosis, and therapeutic endoscopy can be integrated with standard portal hypertension management rather than viewed in isolation [15, 16, 34, 39, 58]. The strength of this field lies not in replacing established tools, but in integrating hemodynamics, fibrosis staging and therapeutic endoscopy into a single decision pathway tailored to the problems that are most characteristic of MASLD. The next challenge is to move from proof of concept to prospective validation showing meaningful impact on treatment allocation, decompensation and patient outcomes. In MASLD, the future role of endohepatology is not to replace existing tools, but to resolve the diagnostic and therapeutic blind spots they leave behind.

## Author Contributions

Writing of the manuscript (M.P.); Critical revision of the manuscript (all authors).

## Funding

The authors have nothing to report.

## Conflicts of Interest

M.P. reports consultant and/or speaker fees from Gore, AstraZeneca, Roche, AbbVie, Gilead, Boston Scientific, MicroTech, Cook Medical, Orphalan, Ipsen, Ewimed and Falk Foundation; no stock ownership; and research funding from the CareerS Clinician Scientist Program and MedK Program (University of Münster), DFG Excellence Cluster EXC2151‐390873048, BONFOR Nachwuchsgruppe 2021‐2A‐07 and the Ernst‐und‐Berta Grimmke Stiftung (Lfd.5/19). W.L. received funding from the MICROB‐PREDICT (project ID 825694), is a co‐holder of the Boston‐Scientific Chair in interventional echoendoscopy and a consultant for Cook Medical, CSL Behring, Boehringer Ingelheim, Roche, Cosmo Health and MRM Health. A.C. has received consulting fees and speaking fees from Boston Scientific Corp and B. Braun and a research grant from Boston Scientific Corp. R.W. received funding from SNF (310030‐189066), Ruth and Arthur Scherbath‐Stiftung and Falk‐Foundation.

## Supporting information


**Figure S1:** Therapeutic endohepatology in acute and definitive management of variceal bleeding. The figure separates initial haemostasis from definitive secondary prophylaxis. In oesophageal variceal bleeding, endoscopic variceal ligation (EVL) is the standard endoscopic approach, followed by combined NSBB plus EVL for secondary prophylaxis; bridging self‐expandable metal stents (SEMS) and TIPS are used in selected cases of treatment failure or high‐risk bleeding. In gastric variceal bleeding, endoscopic cyanoacrylate injection may achieve initial haemostasis, but subsequent definitive management should be selected according to the clinical setting and vascular anatomy. Early TIPS should be considered in eligible high‐risk patients, while EUS‐guided coil‐plus‐cyanoacrylate therapy may provide an alternative when TIPS is contraindicated or technically infeasible. Transvenous obliteration strategies may be considered when an appropriate spontaneous portosystemic shunt is present. BRTO (Balloon‐Occluded Retrograde Transvenous Obliteration), PARTO (Plug‐Assisted Retrograde Transvenous Obliteration), CARTO (Coil‐Assisted Retrograde Transvenous Obliteration), LARTO (Liquid‐Embolic‐Assisted Retrograde Transvenous Obliteration), BATO (Balloon‐Occluded Antegrade Transvenous Obliteration), PTO (Percutanous Transhepatic Obliteration), TACATO (Trans‐Splenic Antegrade Coil‐Assisted Transvenous Obliteration).

**Figure S2:** Therapeutic approaches to gastric varices. The figure illustrates three principal strategies for definitive management of gastric varices. TIPS reduces portal pressure through creation of a transjugular intrahepatic portosystemic shunt and may be combined with variceal embolization. EUS‐guided therapy accesses the variceal complex from the gastrointestinal lumen and permits targeted deployment of coils, cyanoacrylate, or both. BRTO and related transvenous obliteration techniques access the variceal complex through an appropriate spontaneous portosystemic shunt, most commonly a gastrorenal shunt. Transsplenic or transmesenteric access may be considered in selected patients when conventional routes are unavailable or technically unsuitable.

## Data Availability

The authors have nothing to report.
