# Supplementary material for: Endohepatology in the Management of Portal Hypertension in Metabolic Dysfunction‐Associated Steatotic Liver Disease
Source: Liver Int. 2026 Oct 26;46(11):e70953. doi: 10.1111/liv.70953 (PMC13696974; doi:10.1111/liv.70953)
Supplement: Supplementary file 1 [file LIV-46-0-s001.pdf]

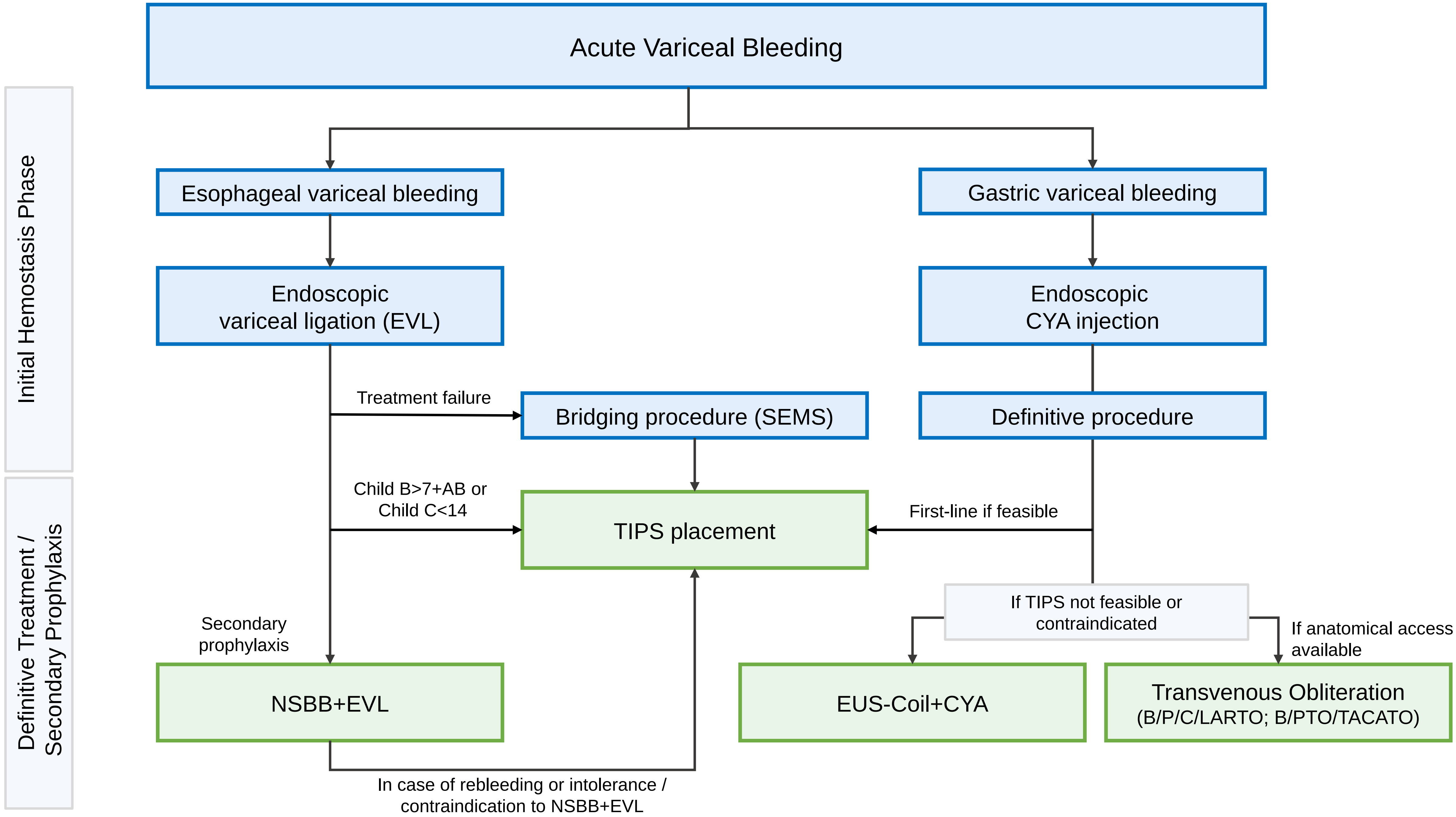
Initial Hemostasis Phase
Definitive Treatment / Secondary Prophylaxis
Acute Variceal Bleeding
Esophageal variceal bleeding
Gastric variceal bleeding
Endoscopic variceal ligation (EVL)
Endoscopic CYA injection
Treatment failure
Bridging procedure (SEMS)
Definitive procedure
Child B>7+AB or Child C<14
TIPS placement
First-line if feasible
If TIPS not feasible or contraindicated
If anatomical access available
Secondary prophylaxis
NSBB+EVL
EUS-Coil+CYA
Transvenous Obliteration (B/P/C/LARTO; B/PTO/TACATO)
In case of rebleeding or intolerance / contraindication to NSBB+EVL
