# Supplementary material for: Endohepatology in the Management of Portal Hypertension in Metabolic Dysfunction‐Associated Steatotic Liver Disease
Source: Liver Int. 2026 Oct 26;46(11):e70953. doi: 10.1111/liv.70953 (PMC13696974; doi:10.1111/liv.70953)
Supplement: Supplementary file 2 [file LIV-46-0-s002.pdf]

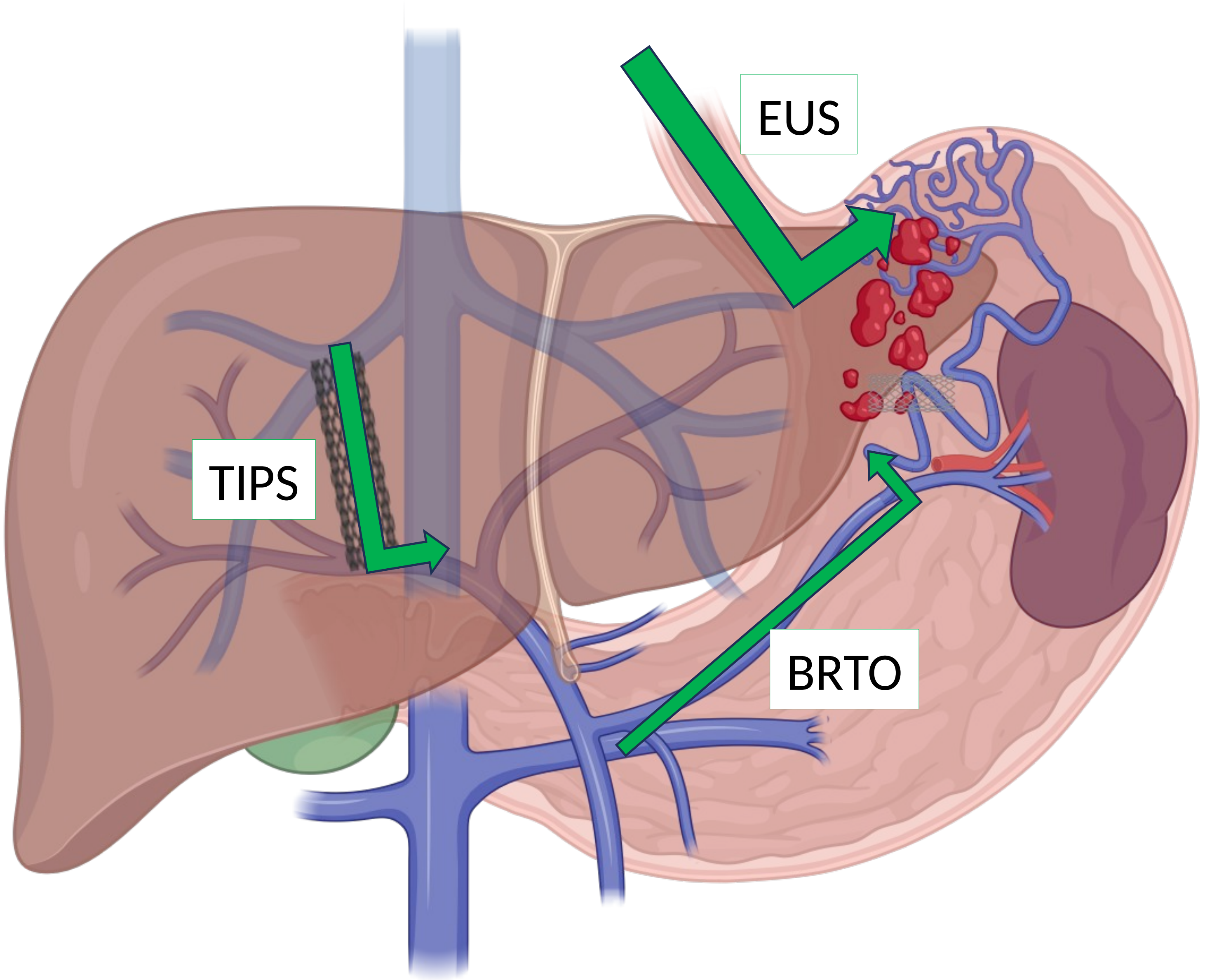
EUS
TIPS
BRTO
